# Detecting Leaf Pulvinar Movements on NDVI Time Series of Desert Trees: A New Approach for Water Stress Detection

**DOI:** 10.1371/journal.pone.0106613

**Published:** 2014-09-04

**Authors:** Roberto O. Chávez, Jan G. P. W. Clevers, Jan Verbesselt, Paulette I. Naulin, Martin Herold

**Affiliations:** 1 Laboratory of Geo-Information Science and Remote Sensing, Wageningen University, Wageningen, The Netherlands; 2 Laboratorio de Biología de Plantas, Departamento Silvicultura y Conservación de la Naturaleza, Universidad de Chile, Santiago, Chile; NASA Jet Propulsion Laboratory, United States of America

## Abstract

Heliotropic leaf movement or leaf ‘solar tracking’ occurs for a wide variety of plants, including many desert species and some crops. This has an important effect on the canopy spectral reflectance as measured from satellites. For this reason, monitoring systems based on spectral vegetation indices, such as the normalized difference vegetation index (NDVI), should account for heliotropic movements when evaluating the health condition of such species. In the hyper-arid Atacama Desert, Northern Chile, we studied seasonal and diurnal variations of MODIS and Landsat NDVI time series of plantation stands of the endemic species *Prosopis tamarugo* Phil., subject to different levels of groundwater depletion. As solar irradiation increased during the day and also during the summer, the paraheliotropic leaves of Tamarugo moved to an erectophile position (parallel to the sun rays) making the NDVI signal to drop. This way, Tamarugo stands with no water stress showed a positive NDVI difference between morning and midday (ΔNDVI_mo-mi_) and between winter and summer (ΔNDVI_W-S_). In this paper, we showed that the ΔNDVI_mo-mi_ of Tamarugo stands can be detected using MODIS Terra and Aqua images, and the ΔNDVI_W-S_ using Landsat or MODIS Terra images. Because pulvinar movement is triggered by changes in cell turgor, the effects of water stress caused by groundwater depletion can be assessed and monitored using ΔNDVI_mo-mi_ and ΔNDVI_W-S_. For an 11-year time series without rainfall events, Landsat ΔNDVI_W-S_ of Tamarugo stands showed a positive linear relationship with cumulative groundwater depletion. We conclude that both ΔNDVI_mo-mi_ and ΔNDVI_W-S_ have potential to detect early water stress of paraheliotropic vegetation.

## Introduction

Heliotropism or ‘solar tracking’ is the ability of many desert plant species and crops to move leaves and flowers as a response to changes in the position of the sun throughout the day [1]. There are two types of heliotropic movements: diaheliotropic movements in which leaves adjust the leaf lamina to face direct solar irradiation, and paraheliotropic movements, in which leaves adjust to avoid facing incoming radiation by contraction of pulvinar structures located at the base of leaves [1]–[3]. Paraheliotropic movements are triggered by directional solar irradiation and allow partial regulation of the incident irradiance on the leaves. For desert species, the regulation of the solar irradiation intensity on the leaves is an important adaptation to avoid photosynthesis saturation (photoinhibition) and to enhance the water use efficiency [4]–[6].

Diurnal paraheliotropic movements have a direct impact on the canopy reflectance properties of vegetation [7]–[9]. Therefore, spectral vegetation indices derived from remote sensing data, like the normalized difference vegetation index (NDVI), can significantly vary during the day and during the year due to leaf movement as solar irradiation changes. Nevertheless, no studies have quantified the effects of solar tracking by plants on the NDVI signal recorded from satellites. Diurnal leaf movements of Tamarugo plants (*Prosopis tamarugo* Phil.) were first described by Chávez et al. [9] under laboratory conditions, and later by Chávez et al. [8] for adult trees in the field. These diurnal leaf movements corresponded to paraheliotropic movements since the leaves moved to an erectophyle leaf distribution (facing away from the sun) around midday when solar irradiation was maximum. The paper of Chávez et al. [8] showed that leaf pulvinar movements caused diurnal changes of Tamarugo's canopy spectral reflectance and NDVI signal, which was negatively correlated to diurnal solar irradiation values.

**Figure 1:**
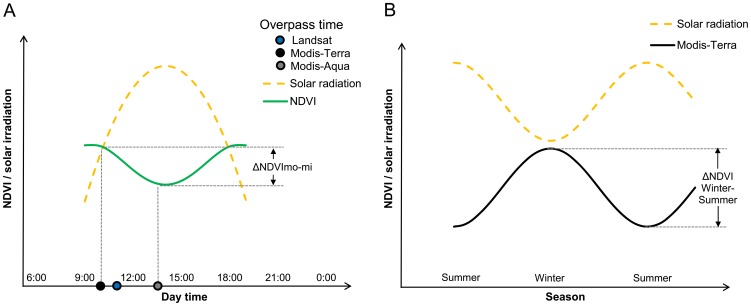
Conceptual diagram of the effect of leaf pulvinar movement on the NDVI signal. (A) NDVI difference between morning and midday (ΔNDVI_mo-mi_) occuring as solar irradiation changes during the day, and (B) NDVI difference between winter and summer (ΔNDVI_W-S_) occuring as solar irradiation varies between seasons. The time at which the Landsat (5–7), MODIS-Terra, and MODIS-Aqua satellites acquire data is displayed to illustrate the impact of pulvinar movements on the NDVI retrieved from these platforms.

In the present study, we hypothesize that the effects of Tamarugo's diurnal leaf pulvinar movements on the NDVI can also be recorded by remote sensors from space, since the acquisition time of the different sensors differ. A high solar irradiation at midday is assumed to cause a lower NDVI than in the morning as indicated in Figure 1a. In this context the MODIS (Moderate Resolution Imaging Spectroradiometer) sensor seems to be especially suitable to capture this difference in NDVI between morning (low solar irradiation, high NDVI) and midday (high solar irradiation, low NDVI), since the MODIS sensor on board of the Terra satellite acquires data for the study site at 10 a.m. (local time) and the MODIS sensor on board of the Aqua satellite acquires data at 1.30 p.m. (local time). Thus, the NDVI difference between morning and midday (ΔNDVI_mo-mi_) can be calculated as the difference between the NDVI MODIS Terra and the NDVI MODIS Aqua.

Considering the negative correlation between diurnal NDVI measurements and solar irradiation reported by Chávez et al. [8], we expect also seasonal NDVI variations associated with seasonal changes in solar irradiation with peaks in winter when the solar irradiation is the lowest. We hypothesize that this effect can also be recorded by sensors from space as indicated in Figure 1b. In this case, the Landsat TM (Thematic Mapper) and ETM (Enhanced Thematic Mapper) catalogue seems to be very suitable for studying NDVI seasonal variations since it offers one of the longest existing time series of systematically recorded satellite data worldwide [10]. Besides, the Landsat catalogue is considered the most relevant satellite dataset for ecological applications and environmental monitoring [11], [12]. MODIS data might also be used to study seasonal effects of the pulvinar movements on the NDVI signal, providing images with a coarser spatial resolution (250 meters vs the 30 meters of Landsat), but with a higher temporal resolution (daily) enabling near real time vegetation monitoring [13]. However, the MODIS time series is considerably shorter than the Landsat time series (only since 2000).

Tamarugo is an endemic tree of the hyper-arid Atacama Desert, Northern Chile, a location considered among the most extreme environments for life [14], [15]. The Tamarugo forest, locally known as Pampa del Tamarugal, sustains a biodiversity of about 40 species of plants and animals, some of them endemic for this particular ecosystem [16]–[19]. Precipitation events are very rare and the only source of water supply for vegetation is the groundwater (GW), from which Tamarugo is completely dependent. However, not only Tamarugo trees are demanding water: the main economic activity in Atacama is mining, which is also demanding water for human consumption and for many industrial processes. This has led to an overexploitation of the GW sources and a progressive depletion of the GW over the whole Pampa del Tamarugal [20].

The natural Tamarugo forest was almost extinct in the 19^th^ century and during the 1970's an enormous reforestation effort was carried out by the Chilean government and 13,000 hectares of Tamarugo were planted in the Pampa del Tamarugal basin [21]. Currently, the Pampa del Tamarugal is under threat due to GW overexploitation. Chilean policy makers, scientists and private companies have debated intensively about defining environmentally safe GW extractions. To achieve this, good indicators of the Tamarugo water condition are needed and remote sensing, and specifically the NDVI, has proved to be useful for assessing Tamarugo's water condition [8], [9]. Nevertheless, time series of NDVI have not been directly related to GW depletion yet and to do so, the effect of the leaf pulvinar movements must be considered to understand a) the natural NDVI dynamic in the absence of water stress, and b) how this dynamic may be altered by GW depletion. In this paper, we use MODIS and Landsat NDVI time series to study both the natural and the altered NDVI dynamics of Tamarugo stands located in the Pampa del Tamarugal basin. Furthermore, we explore other biological (phenology) and environmental factors (precipitation) with potential effects on the NDVI signal.

## Material and methods

### 2.1 Species description

**Figure 2:**
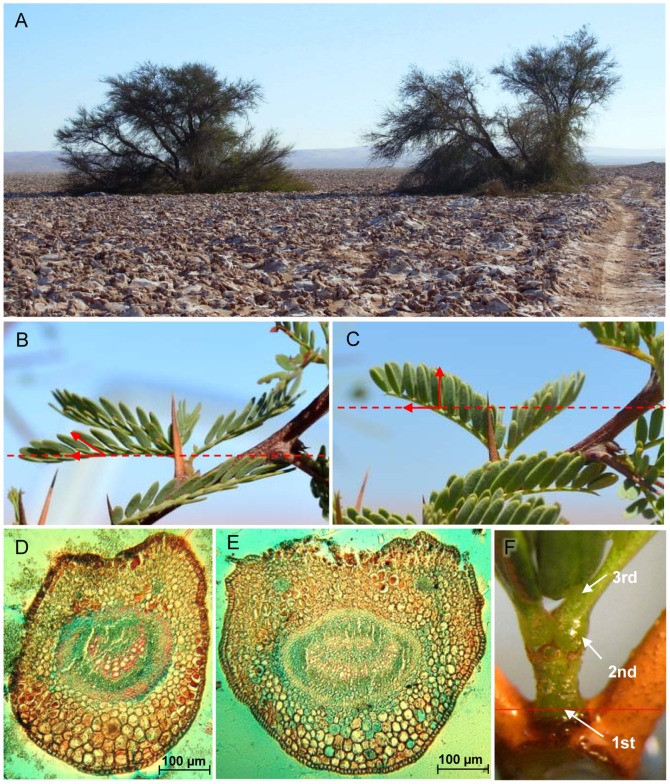
Pulvinar structures of Prosopis tamarugo leaves. (A) Tamarugo trees, (B) leaf angle randomly distributed during the morning when the solar radiation is low, (C) leaf angle in erectophyle position to avoid facing high solar irradiation at midday, (D) transversal section of a closed pulvinus (empty of water) during the morning, (E) transversal section of an open pulvinus (filled with water), which allows leaves to stand up and reach the erectophyle position, and (F) detail of the base of a Tamrugo pinna showing the three levels of pulvinar structures (at the base of the bipinna, of each pinna and each foliole).

Tamarugo is a phreatophytic desert tree that is highly specialized to survive the hyper-arid conditions of the Atacama Desert. This species belongs to the Leguminoseae family, Mimosaceae subfamily and it can reach up to 25 meters height, 20–30 meters crown size and 2 meters stem diameter [22], [23]. The branches are arched and twigs flexuous with composite leaves, often bipinnate with 6–15 pairs of folioles (Figure 2b, c, f) [24]. The Tamarugo petioles have a distinctive structure of motor cells in the pulvinus, responsible for the leaf paraheliotropic movements (Figure 2d, e, f). Differential turgor changes of the pulvinus cells make the leaves to stand up and orientate the leaf lamina parallel to the incoming sun rays. The composite leaves of Tamarugo have three levels of pulvinar structures: the first at the base of the bipinna, the second at the base of each pinna, and the third at the base of each of the folioles. This pulvinar mechanism at the three levels allows the Tamarugo canopy to adjust its internal structure to avoid facing excessive solar irradiation. Tamarugos are phreatophytic species [25], [26] presenting a dual root system consisting of a deep taping root and a dense superficial root mat [27]. This dual system would allow Tamarugos to move water from the deep groundwater table to the superficial root mat layer during the night to ensure water supply during the growing season when the water demand at the capillary fringe increases [26].

### 2.2 Study area

**Figure 3:**
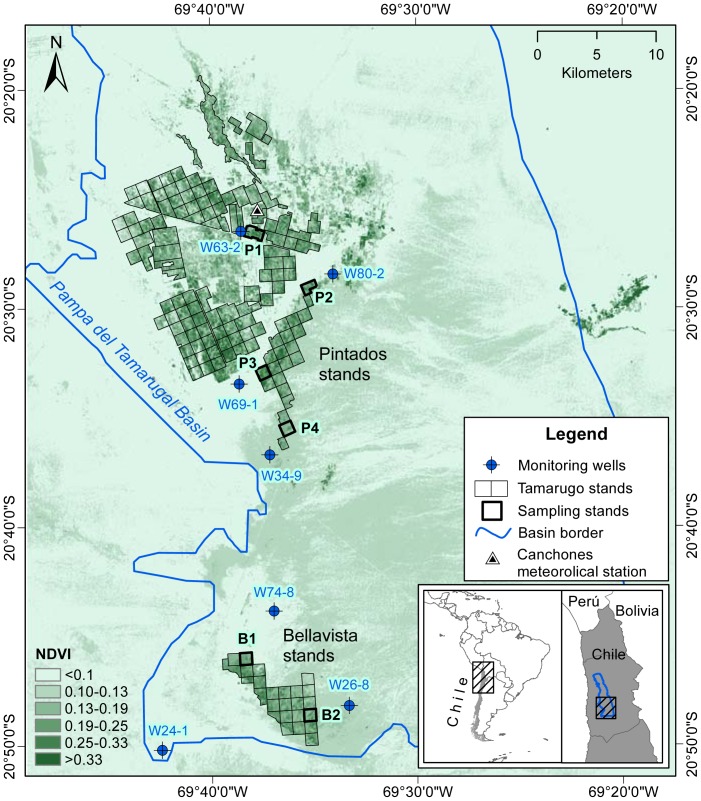
Landsat NDVI image showing the location of the Tamarugo stands (Winter 2007).

The study area is located in the Atacama Desert (Northern Chile), specifically in the southern part of the Pampa del Tamarugal basin, where most of the remaining Tamarugo population is concentrated (Figure 3). The Tamarugo forest is practically the only ecosystem of the Absolute Desert eco-region [16], and it is characterized by almost null precipitation, high day-night temperature oscillation, and high potential evapotranspiration [28], [29]. Most of the plantation stands (Pintados and Bellavista) are in the southern part of the basin and within the study area. Just little natural patches of Tamarugo remain in the northern portion of the Pintados plantation (Figure 3). Although the oldest plantation stands were established as early as 1936, most of the existing plantation stands were planted between 1968 and 1972 [30]. The plantation scheme consisted of squared 1×1 kilometres stands and trees separated 10×10 meters. Besides Tamarugo plantations, there are plantations of other *Prosopis* species, sometimes mixed with Tamarugo. Only pure Tamarugo plantation stands and some natural forest patches were considered in this study and they can be identified in Figure 3 as the green areas highlighted in black.

### 2.3 Landsat and MODIS NDVI time series

We used all available Landsat 5 TM and Landsat 7 ETM data (referred from here onwards in the text as ‘Landsat’ data) as well as MODIS-Terra and MODIS-Aqua data of the study area covering the period 1989–2012. We selected this time frame since this is the period of time with available GW depth records for most of the monitoring wells located in the study area (Figure 3). For the Landsat NDVI time series we used cloud free L1T images of 30 meters pixel resolution (471 scenes) corresponding to path 1 and row 34 and pre-processed using the Landsat Ecosystem Disturbance Adaptive Processing System (LEDAPS) to obtain surface reflectance values for all spectral bands [31]. Finally, we used the surface reflectance values of red and NIR to compute the NDVI for each date as follows: NDVI  =  (NIR-Red)/(NIR+Red). For the MODIS-Terra and MODIS-Aqua NDVI time series we used the MODIS 16-day composites at 250 meters pixel resolution (MOD13Q1 and MYD13Q1 data products). MODIS pixel reliability showed that 85% of the observations can be used with confidence (reliability  = 0) and 15% were considered useful (reliability = 1) of which MODIS vegetation index quality indicated average aerosol quantity. MODIS pixels with reliability 0 and 1 were considered in this study and showed consistent values for the NDVI time series of all forest stands. Both MODIS and Landsat data were downloaded from the USGS Earth Explorer website. Complementary, we used a panchromatic WorldView2 image of 0.6 meters pixel resolution to quantify the tree coverage of each plantation stand. This was carried out by using object-based image classification and the eCognition software following the procedure used by Chávez et al. [8].

### 2.4 Groundwater and climatic data

GW records were obtained from the monitoring network of the Dirección General de Aguas (DGA), the Chilean Water Service. From this network, seven wells were close to the Tamarugo stands and had enough records to establish a direct relationship between the groundwater table and the forest status (Figure 3). We averaged the (three to twelve) records of each year to obtain annual values of groundwater depth for the seven monitoring wells. Figure 3 shows the location of the monitoring wells used in this study as well as the forest stands located close to each well. Basic data of each monitoring well are provided in Table 1. This way we obtained representative groundwater data for six Tamarugo stands for the period 1989–2012. In the case of stands B1 and B2, the groundwater depth was estimated using an inverse distance weighted interpolation of records from three wells (see Table 1).

**Table 1:** Plantation stands close to monitoring wells in the Pampa del Tamarugal basin.

					Groundwater depth (m)			
Stand	Plantation year	Canopy coverage (%)	Closest monitoring well (DGA code)	Distance to well (km)	1989	1997	2007	2012
B1	1968–1969	17	017000-74-8	4.7	10.99*	11.19*	11.64*	11.90*
			017000-26-8	9.5				
			017000-24-1	10.1				
B2	1968–1969	21	017000-74-8	9.3	14.75*	14.87*	15.45*	15.82*
			017000-26-8	3.4				
			017000-24-1	12.8				
P1	unknown	22	017000-63-2	0.4	7.97	9.03	9.93	10.26
P2	1972	25	017000-80-2	1.6	9.90	11.91	12.97	13.11
P3	1972	27	017000-69-1	1.7	5.70	6.26	6.87	6.89
P4	1972	11	017000-34-9	1.9	7.93	8.65	9.54	9.55

(*)Groundwater depth and depletion estimated using inverse distance weighted interpolation of 3 neighbouring wells.

Although groundwater is the main water source of the Tamarugo forest, sporadic precipitation may occur in the Atacama Desert, having a positive impact on the water status of the trees and the NDVI signal. For this reason, we included in our analysis precipitation records from the DGA meteorological station Huara en Fuerte Baquedano (20°07'51''S, 69°44'59''W) located about 30 km north from the study area and at a similar altitude (1,100 m). Solar irradiation records were obtained from the Canchones Experimental Station of the Universidad Arturo Prat (Chile), located next to the Tamarugo stand P1 in the northern part of the study area (Figure 3).

### 2.5 Data analysis

#### 2.5.1 NDVI signal in the absence of water stress (natural dynamic)

Finding Tamarugo vegetation without nearby GW depletion in the Atacama Desert was a difficult task. We identified a Tamarugo forest stand (B1) and a time frame (2005-2008) with almost null GW depletion and no precipitation events in the southern part of the study area (see Figure 3, Bellavista stand). We assumed the NDVI time series of this three year period was not strongly influenced by the growth of trees. The Landsat, MODIS-Terra and MODIS-Aqua NDVI time series for the stand B1 were calculated using the median value of the pixels inside the 1×1 km stand. This aggregation enabled direct comparison of Landsat and MODIS NDVI time series. In the case of the Landsat time series we excluded pixels with NDVI values lower than 0.13, which were considered as no forest pixels. This threshold was set by considering the NDVI values observed outside the plantation stands, which correspond to completely bare areas (Figure 3). We first analysed the time series without any level of temporal aggregation, and then we aggregated the values to monthly averages in order to study the relationship between the NDVI and the monthly mean solar irradiation. For the latter purpose we used simple linear regression.

#### 2.5.2 NDVI signal under water stress

After studying the natural dynamic of the NDVI time series for the three satellite sensors, we analysed the relationship between the average annual records of GW depletion and different metrics derived from the NDVI signal. To achieve this we used simple linear regression between the cumulative GW depletion and the NDVI derived metrics of the period between 1997 and 2007 with no precipitation. For the Landsat NDVI time series, these metrics were: annual NDVI average (NDVI_av_), NDVI in winter (NDVI_W_), and the NDVI difference between winter and summer (ΔNDVI_W-S_). For MODIS NDVI time series, these metrics were the ΔNDVI_W-S_ and the NDVI difference between morning and midday (ΔNDVI_mo-mi_). We calculated ΔNDVI_W-S_ and ΔNDVI_mo-mi_ for each year as follows:

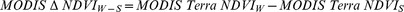
,

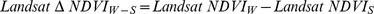
, and

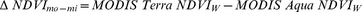

Where: 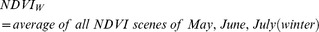


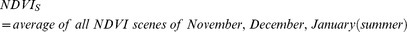



In the case of the Landsat NDVI time series, we considered a minimum of three scenes for the summer and winter period to obtain a representative value of the respective season. For all Landsat and MODIS NDVI scenes we used the median value of the pixels inside the 1×1 km stands.

## Results

### 3.1 Leaf pulvinar movement and the NDVI natural dynamic

**Figure 4:**
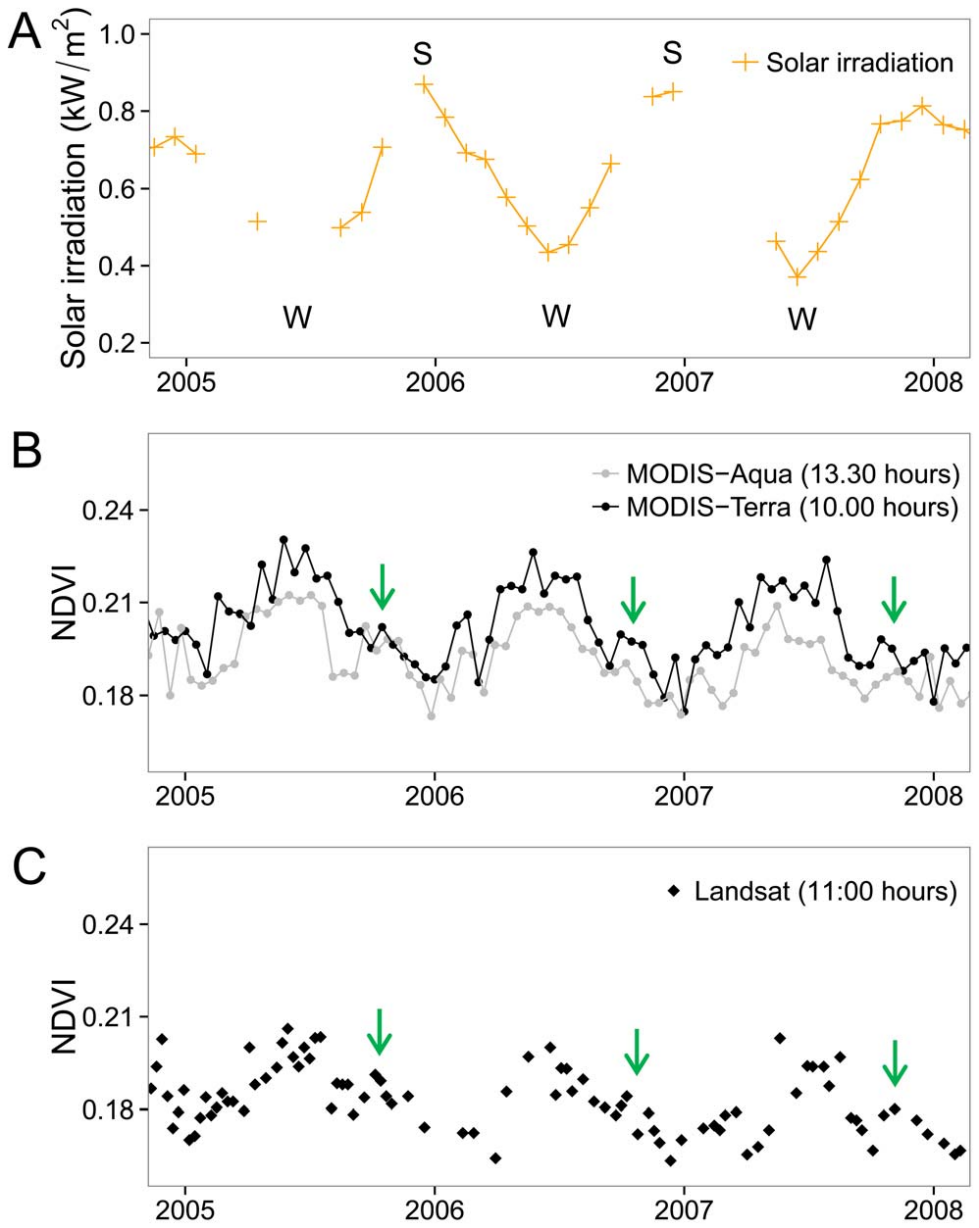
Time series of solar irradiation and NDVI for the B1 site (low groundwater depletion). (A) Solar irradiation, (B) MODIS 16 days composite NDVI, and (C) Landsat NDVI of the B1 site. Arrows indicate the peak of Tamarugo's vegetative period. S = summer, W = winter.

In the absence of GW extraction or precipitation events, the NDVI signal of the Tamarugo stand B1 presented a strong seasonal variation for the period 2005–2008, mainly explained by the seasonal variation of the monthly average solar irradiation (Figure 4) influencing the pulvinar movement of paraheliotropic plants (Figure 1). The R^2^ for the linear relationship between NDVI and solar irradiation was 0.66 for the Landsat NDVI time series, 0.65 for the MODIS-Terra NDVI, and 0.41 for the MODIS-Aqua NDVI. Partial foliage loss during the period May-September and the peak of the vegetative period occurring around October seemed to have only a marginal effect on the NDVI time series, noticeable as a small drop followed by a peak around October (green arrows in Figure 4). Overall, the seasonal variation is the main feature of the annual NDVI signal, and therefore the ΔNDVI_W-S_ may be used to detect the leaf pulvinar movement occurring in the Tamarugo canopy under natural conditions.

**Figure 5:**
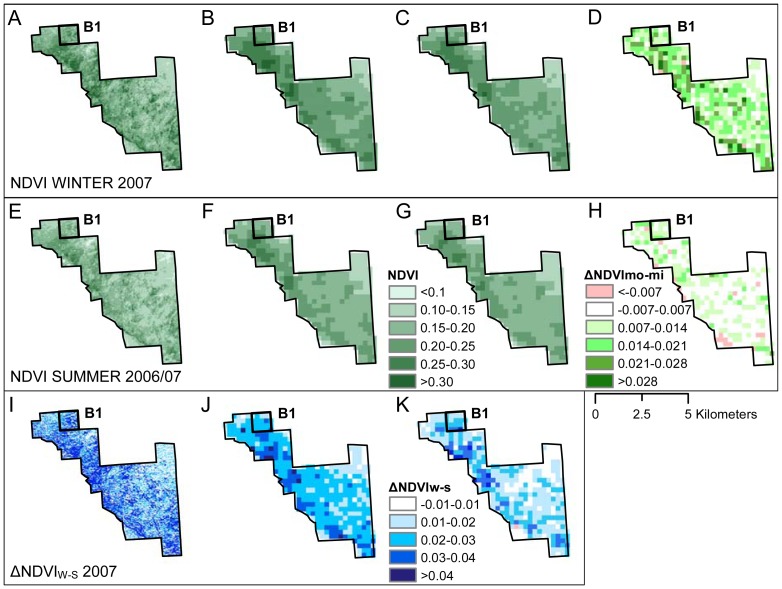
ΔNDVI morning-midday and ΔNDVI winter-summer of the Bellavista plantation in 2007. **Winter 2007:** (A) Landsat NDVI, (B) MODIS-Terra NDVI (morning), (C) MODIS-Aqua NDVI (midday), (D) ΔNDVI_mo-mi_  =  B–C; **Summer 2006–07**: (E) Landsat NDVI, (F) MODIS-Terra NDVI (morning), (G) MODIS-Aqua NDVI (midday), and (H) ΔNDVI_mo-mi_  =  F–G. Graphs I, J and K display the **ΔNDVI_W-S_ 2007**, where (I) Landsat ΔNDVI_W-S_  =  A–E, (J) MODIS-Terra ΔNDVI_W-S_  =  B–F, and (K) the MODIS-Terra ΔNDVI_W-S_  =  C–G.

Besides the seasonal variation, the MODIS-Terra and MODIS-Aqua NDVI time series allowed to identify the ΔNDVI_mo-mi_ reported by Chávez et al. [8] on single Tamarugo trees, for instance for the Tamarugo stand B1 (Figure 4b). Although the ΔNDVI_mo-mi_ was clearly noticeable during winter, it was close to zero in summer. This was expected since both the morning and midday solar irradiation in the Atacama Desert are much higher in summer than in winter. For example, the average solar irradiation of June 2007 (winter) was 0.21 kW/m^2^ at 10.00 hours and 0.62 kW/m^2^ at 13.30 hours while the average of December 2006 (summer) was 0.87 kW/m^2^ at 10.00 hours and 0.94 kW/m^2^ at 13.30 hours. As a result, in winter the leaves will only have an erectophile position at midday, but in summer this occurs already half way the morning (yielding a small ΔNDVI_mo-mi_). Based on what we observed in Figure 4 for a Tamarugo stand without water stress we can expect that it has a positive ΔNDVI_mo-mi_ in winter as well as a positive ΔNDVI_W-S_. Both NDVI derived metrics can be quantified and mapped using Landsat and MODIS images as shown in Figure 5.

Figure 5 displays the NDVI values at pixel level of all Bellavista plantation stands (including the stand B1) in the winter of 2007 (first row), the summer of 2006–2007 (second row), and the ΔNDVI_W-S_ of 2007 (third row) obtained from Landsat images (first column), MODIS-Terra images (second column), and MODIS-Aqua images (third column). The fourth column corresponds to the ΔNDVI_mo-mi_ in winter (Figure 5d) and summer (Figure 5h) based on Terra (morning) and Aqua (midday). This figure confirms that the ΔNDVI_mo-mi_ in winter and the ΔNDVI_W-S_ of 2007 was positive for the forested area. On the other hand, the ΔNDVI_mo-mi_ in summer was zero or close to zero. In a similar way, and as a consequence of the diurnal pulvinar movements, the ΔNDVI_W-S_ was higher when using MODIS-Terra images than when using MODIS-Aqua images. Thus, the most promising indicators of pulvinar movement seemed to be the ΔNDVI_mo-mi_ in winter and the ΔNDVI_W-S_ in the morning (MODIS-Terra). When using the NDVI as a potential indicator of Tamarugo's water status, the signal in winter was stronger. The canopy coverage can also play an important role in the strength of the NDVI signal and its effect has to be considered when using these NDVI derived metrics for monitoring purposes. We will discuss this issue further in the next section where more Tamarugo stands, with different canopy coverage, were analysed.

### 3.2 Groundwater depletion: the NDVI signal under water stress

**Figure 6:**
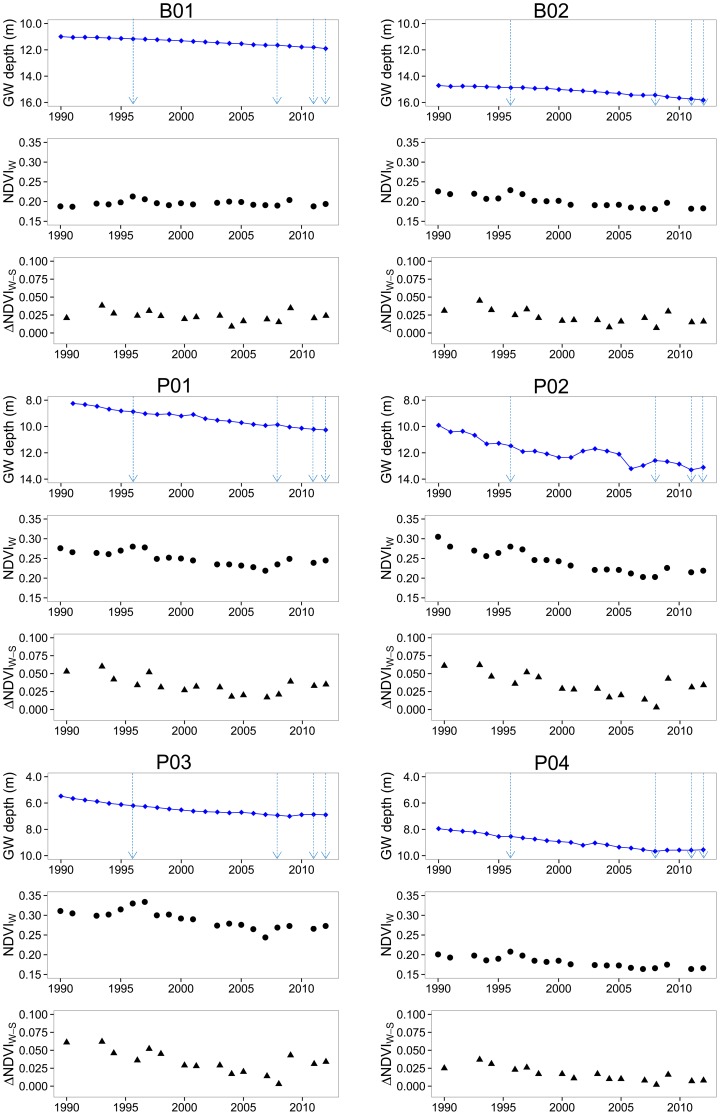
Time series of groundwater depth, Landsat NDVI_W_, and Landsat ΔNDVI_W-S_ for six Tamarugo plantation stands. Blue arrows indicate precipitation events.

Figure 6 displays the annual time series of GW depth and the Landsat NDVI_W_ and ΔNDVI_W-S_ for the six Tamarugo stands analysed in this study. The precipitation events are indicated with arrows. Only four precipitation events were recorded in the 24 years period analysed: 3.0 mm in 1996, 1.8 mm in 2008, 7.9 mm in 2011, and 2.2 mm in 2012, three of them during the last five years. The Landsat NDVI_W_ signal reacted to the precipitation event of 1996 by showing a short recovering phase (about one year) and quickly returned to the general decreasing trend. For the precipitation events in the last years this effect was difficult to observe since they occurred close to each other in time. These precipitation events did not have any impact on the groundwater table, so we assumed this water was only available for the trees in the superficial soil layers. Although these precipitation events contributed little water to the basin, we assume the moisture added to the superficial root mat of Tamarugo trees temporally had a positive impact on the growth of the trees. Apart from the precipitation events, the analysed NDVI metrics seemed to follow the GW depth trend for all stands. To quantify this relationship, we calculated the R^2^ for the linear regression between each of the NDVI metrics and the cumulative GW depletion for the period without precipitation (1997–2007). We also included in this analysis the Landsat annual NDVI_av_ values to check whether the NDVI_W_ was a better indicator than the simple annual NDVI average. The results are given in Table 2.

**Table 2:** R^2^ of the linear model of cumulative groundwater depletion v/s NDVI_av_, NDVI_W_, ΔNDVI_W-S_ and ΔNDVI_mo-mi_ for the period 1997–2007 (no precipitation events).

Stand	Cumulative GW depletion (1997–2007)	Landsat NDVI_av_	Landsat NDVI_W_	Landsat ΔNDVI_W-S_	MODIS ΔNDVI_W-S_	MODIS ΔNDVI_mo-mi_
		n = 11	n = 10	n = 8	n = 4	n = 5
B1	0.45	<0.1	0.13	0.44*	0.55	0.29
B2	0.58	0.74***	0.76***	0.26	0.76	0.35
P1	0.90	0.58***	0.75***	0.60**	<0.1	<0.1
P2	1.06	0.27*	0.29	0.24	0.70	0.70
P3	0.61	0.82***	0.90***	0.70***	0.66	0.14
P4	0.89	0.77***	0.85***	0.74***	<0.1	0.52

Significative linear relationship with ***P<0,01; **P<0.05, *P<0.1.

av  =  average; W  =  winter; W-S  =  winter-summer; mo-mi  =  morning-midday.

The Bellavista Tamarugo stands (B1 and B2) are located in the southern part of the basin and far from the area where the pumping wells are concentrated, which is towards the north and east of the Pintados stands (Figure 3). For this reason, the GW depletion in the stands B1 and B2 was less in comparison to the stands of the Pintados sector (P stands), especially in the case of P2. The stand B1 showed the lowest cumulative depletion (0.45 m) for the period 1997–2007 as well as the lowest R^2^ (<0.1) for the relationship between Landsat NDVI_av_ and GW depletion. Furthermore, the R^2^ of the GW depletion - Landsat NDVI_W_ relationship was also the lowest, but higher than the GW depletion - Landsat NDVI_av_ relationship. In fact, this was the case for almost all stands. Thus, the Landsat NDVI_W_ was more sensitive to changes in GW depth than the Landsat NDVI_av_. This was also the case when comparing Landsat NDVI_W_ with Landsat ΔNDVI_W-S_. Only for the stand B1, the R^2^ of the Landsat ΔNDVI_W-S_ - GW depletion relationship was higher than for the NDVI_W_ - GW depletion relationship.

The rest of the stands showed GW depletions between 0.58 and 1.06 meters between 1997 and 2007 and R^2^ values for the Landsat NDVI_W_ - GW depletion relationship higher than 0.75 except for the stand P2 with an R^2^ of 0.29. The stand P2 is located close to the pumping area, and therefore the GW depletion could have been influenced by short-term changes of the pumping rate. If the intra-annual GW values fluctuated too rapidly, the depletion may not have had an effect on the NDVI signal. However, this is difficult to detect in annually averaged records. Overall the Landsat NDVI_W_ was the most sensitive NDVI derived metric to the 11-year changes in GW depletion.

In the case of the MODIS NDVI derived metrics, the R^2^ values presented in Table 2 were difficult to interpret since the time series without precipitation events was very short (2003–2007). We found R^2^ values as high as 0.70 when using the MODIS ΔNDVI_W-S_ (stand B2) and the MODIS ΔNDVI_mo-mi_ (stand P2), but also <0.1 (stands P1 and P4) for ΔNDVI_W-S_ or ΔNDVI_mo-mi_ (stand P3).

### 3.3 Mapping water stress using Landsat ΔNDVI_W-S_

**Figure 7:**
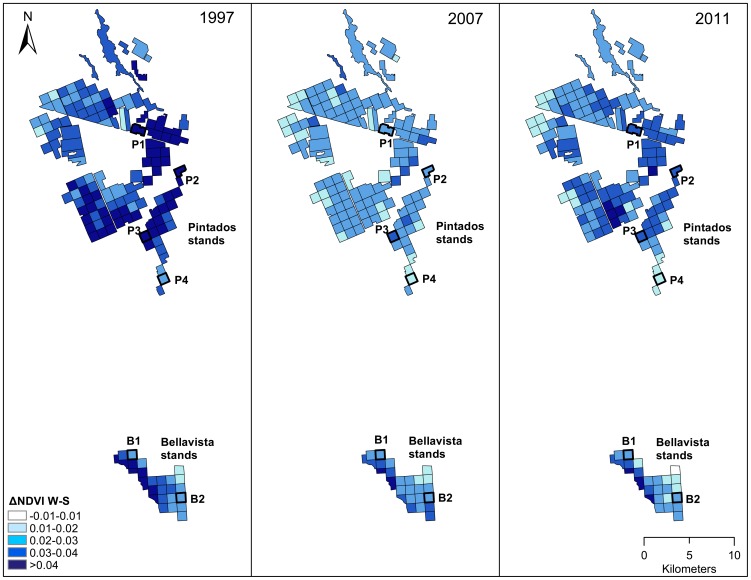
Landsat ΔNDVI_W-S_ of all plantation stands in 1997, 2007, and 2011 (after a precipitation event).

The NDVI_W_ and ΔNDVI_W-S_ showed good potential to assess the effect of GW depletion on the water status of Tamarugo trees. We selected the Landsat ΔNDVI_W-S_ to map this effect in the study area because we believe it senses water stress earlier than NDVIw (see Discussion section for more details). We mapped the ΔNDVI_W-S_ for three different years: 1997, 2007, and 2011 as shown in Figure 7 averaged to a 1×1 km grid. The ΔNDVI_W-S_ difference between 1997 and 2007 can be explained by groundwater depletion since no precipitation events occurred in this period. For most of the stands, the ΔNDVI_W-S_ values in 2011 showed a recovery of the forest after the precipitation event of 2011 (7.9 mm), the most intense rain recorded in the last 25 years in Pampa del Tamarugal. The stands with more stable ΔNDVI_W-S_ through time were those located at the west border of the Bellavista plantation, close to the well W24-1, which reported very shallow GW depths in 1997 (2.6 m), 2007 (3.0 m), and 2011 (3.1 m). Furthermore, a ΔNDVI_W-S_ gradient can be observed in the Bellavista sector from east to west, showing a good spatial agreement with the increasing GW depletion towards the east (GW depth in well W26-8 was about 19.6 m in 1997, 20.3 m in 2007, and 21 m in 2011).

## Discussion

Early stages of water stress in plants are associated with a lower leaf water potential, reduction in transpiration rate, and foliage water loss while late stages are associated with pigment degradation, biomass loss, and finally dying plants [32]–[34]. Although Tamarugo trees are naturally adapted to the predominant water scarcity of the Atacama desert, they can be affected by water stress due to GW depletion as shown in this paper. From previous papers [8], [9], we know that Tamarugos show the typical water stress symptoms of most plants, but additionally water stress limits the normal functioning of the leaf pulvinar mechanism. These pulvinar movements are typical for heliotropic species. Furthermore, we have shown that leaf pulvinar movement can be remotely sensed by different metrics derived from the NDVI signal, allowing to understand both the temporal natural dynamic of the Tamarugo forest and the effects of GW depletion. In Table 3 we give an overview of the water stress symptoms of Tamarugo trees, the temporal scale at which they occur, and the NDVI derived metrics we can use to study these symptoms.

**Table 3:** Water stress stages of Tamarugo desert trees and NDVI based variables to assess their effects using satellite remote sensing.

Water stress			Remote sensing	
Stage	Effects on Tamarugo vegetation	Temporal scale to perceive the effects	Monitoring variable	Sensor useful to retrieve this variable
Instantaneous	Limitation of the diurnal photoinhibition control via pulvinar movements	Diurnal	ΔNDVI_mo-mi_	MODIS Terra and Aqua
Early	Limitation of the seasonal photoinhibition control via pulvinar movements	Seasonal	ΔNDVI_W-S_	MODIS Terra; Landsat
Advanced	Foliage loss	More than 1 year	NDVI_W_	MODIS Terra; Landsat
Irreversible	Partial crown death and tree death	Several years	NDVI_W_	MODIS Terra; Landsat, combined with very high spatial resolution sensors: Quickbird2, WorldView2 or GeoEye

Diurnal leaf movements can be studied using ΔNDVI_mo-mi_ from MODIS Terra and Aqua satellites as shown in this paper. No significant differences have been found for the MODIS NDVI Terra and Aqua for other non-solar tracker vegetation [35], [36]. Since these two satellites acquire data on a daily basis, it would be possible to map the ΔNDVI_mo-mi_ of the Pampa del Tamarugal basin every day at a spatial resolution of 250×250 m. This way, the effects of an abrupt GW depletion could be identified using MODIS data if the forest is dense enough to provide a sufficiently strong signal as well as large enough to cover one or more MODIS pixels [37], [38]. In this paper we analysed averaged ΔNDVI_mo-mi_ values for the winter seasons and its relationship with annual records of GW depth. This time series was rather short, sometimes resulting in low R^2^ values. Perhaps better results can be achieved when using the full temporal resolution (daily or 16 days) of the MODIS NDVI products and more detailed records of the water availability. This is an interesting topic for further research and not only for Tamarugo plants, but also for detecting short-term water stress in, e.g., bean crops, which also have documented paraheliotropic behaviour [5], [6].

Seasonal differences of leaf pulvinar adjustments of Tamarugo vegetation can be studied at a large scale using the ΔNDVI_W-S_ as measured from Landsat (Figure 7) and MODIS Terra satellites. The advantage of using Landsat images is the possibility to map this variable at 30 meters pixel resolution and the disadvantage is that these satellites (Landsat 5, 7 and 8) have a revisit time of 16 days, increasing the chance of missing dates due to cloud cover. Although cloud cover is not such as problem in deserts, missing data can have an important impact on the calculation of the ΔNDVI_W-S_ if the NDVI values of winter or summer are not well represented by sufficient images. In this paper, we considered a minimum of three Landsat scenes for calculating a representative value of the summer or winter period. The NDVI signal of Tamarugo showed a strong seasonality (Figure 4c) and, for example, a calculation of the NDVI_W_ using one or two images in May and a calculation of the NDVI_S_ using one or two images in December may lead to a serious underestimation of the ΔNDVI_W-S_. This is not a problem for MODIS 16-day composites, which provide five or six images for the winter and summer period systematically distributed within the three months' timeframe. Therefore, there is a trade-off between temporal and spatial resolution when choosing Landsat or MODIS to detect the ΔNDVI_W-S_.

If the water stress persists, Tamarugo trees will react by selectively shutting down leaves, twigs and entire branches to reduce the transpiration surface while keeping the remaining foliage green with hydric parameters within normal ranges [8]. Foliage loss has been successfully assessed using NDVI for a wide range of vegetation types and it is especially accurate for LAI values <2 [39]. Such assessments are usually carried out at the peak of the vegetative period, usually in spring. In the case of Tamarugo the seasonal variation of the NDVI signal is mainly driven by the pulvinar movements, which are primarily driven by seasonal changes in solar irradiation. Thus, the ‘pulvinar effect’ on the NDVI signal is minimum in winter and therefore this is the best time to retrieve the NDVI for inter annual foliage loss estimations (Table 3).

The strong relationship between NDVI_W_ and cumulative GW depletion observed for most of the Tamarugo stands is an indication that foliage is decreasing in the study area as a consequence of water extraction, in other words, the forest is reaching an advanced stage of water stress (Table 3). However, it was not possible to discriminate whether the decreasing NDVI_W_ signal was because some trees were dying while others remained alive (intra species competition) or all trees were losing foliage gradually. The tree coverage played also an important role in the absolute value of the NDVI_W_ signal and, therefore, it was not possible to directly compare different stands at a single point in time. In order to better interpret the Landat and MODIS NDVI_W_ signal, we believe that high spatial resolution remote sensing data can provide complementary information about the actual tree coverage of the forest as well as the water status of single trees. This will be the topic for further research.

A recent publication entitled ‘Remote sensing: A green illusion’ [40] has drawn the attention of the scientific community and policy makers on the issue of the correct interpretation of remote sensing derived products for environmental applications. The authors reflected on this issue based on the results of Morton et al. [41] showing how the apparent canopy greenness of the Amazon forest, interpreted as a positive response to more sunlight in the dry season, was caused by a bidirectional reflectance effect. In other words, it was caused by an optical artefact due to seasonal changes of the sun-sensor geometry. In this paper, we also discussed the correct interpretation of remote sensing derived products, but this time for paraheliotropic vegetation. As shown for the case of Tamarugo in this study, the seasonal changes in NDVI were related to leaf pulvinar movements causing a change in the canopy structure. This change in canopy structure explained the observed seasonal changes (Figure 4). Three pieces of evidence support the hypothesis that pulvinar movements are responsible for NDVI diurnal and seasonal changes of Tamarugo vegetation and that this is not an optical artefact due to bidirectional reflectance effects:

- As shown in a previous paper [9], canopy spectral reflectance of Tamarugo plants simulated with the Soil-Leaf-Canopy (SLC) radiative transfer model showed that the SLC parameter LIDF (leaf inclination distribution function) could explain diurnal changes in canopy reflectance measured empirically with a spectroradiometer under laboratory conditions (lamp-sensor geometry was fixed). Thus, leaf movements, set in the SLC simulations as a ‘random’ LIDF in the morning and as an ‘erectophile’ LIDF after midday, explained diurnal changes in canopy reflectance in the absence of water stress.
- Another previous paper [8] showed a negative empirical relationship between diurnal values of NDVI, measured for single Tamarugo trees with a spectroradiometer, and solar irradiation under field conditions. In that study, the authors observed a predominantly erectophyle position of Tamarugo leaves around midday, corresponding to the diurnal peak of solar irradiation and the lowest values of NDVI. In the current paper, we showed a negative empirical relationship between seasonal NDVI values, measured by Landsat and MODIS satellites for Tamarugo stands, and solar irradiation (Figure 4). Furthermore, it is a known botanical fact that paraheliotropic movements are a response to increasing solar irradiation on the leaves [1]. Thus, pulvinar movements activated by changes in solar irradiation govern diurnal and seasonal changes in the NDVI signal of Tamarugo vegetation.
- This study provided evidence that the amplitude of the seasonal NDVI trend (ΔNDVI_W-S_) of Tamarugo stands declined with water stress (Figure 6). If the NDVI seasonal trend measured by satellite remote sensing was governed by a sun-sensor artefact, there is no reason why water stress would cause the amplitude of the NDVI signal to decline significantly.

In the southern hemisphere, more internal shadowing in satellite images (captured at nadir) is expected to occur in winter at lower solar elevation, and therefore, the bidirectional reflectance effect should cause an ‘apparent greening’ towards spring/summer [41]. However, as shown in Figure 4, the peak of the NDVI signal of Tamarugo stands does not occur in summer, but in winter. Although bidirectional reflectance effects may also occur in the case of Tamarugo vegetation, we believe that such effects are obscured by the stronger effect of seasonal pulvinar movement.

## Conclusions

1. Monthly values of solar irradiation were negatively correlated to NDVI measured by the MODIS-Terra and Landsat satellites. Previous studies have shown that pulvinar movement causes the NDVI signal to drop from morning to midday as solar irradiation increases, and therefore, in the absence of water stress the seasonal variation of NDVI is also expected to be controlled by pulvinar movement.
2. The NDVI difference between midday and morning (ΔNDVI_mo-mi_), as measured by the difference of the NDVI signal from the MODIS Terra and Aqua satellites, can be used to detect the diurnal leaf pulvinar movement of Tamarugo plantation stands. This has not been reported in literature before, and therefore, this paper constitutes a proof of concept that MODIS images can be used to detect diurnal movements of paraheliotropic vegetation.
3. Similarly, the NDVI difference between winter and summer (ΔNDVI_W-S_), as measured by the Landsat or the MODIS Terra satellites, can be used to detect differences in seasonal pulvinar movements, associated to photoinhibition regulation.
4. Leaf pulvinar movements are triggered by changes in cell turgor and they can be limited by water stress. Thus, water stress in Tamarugo vegetation caused by groundwater overexploitation can be assessed and monitored using ΔNDVI_mo-mi_ and ΔNDVI_W-S_. For long time series (more than 10 years), Landsat ΔNDVI_W-S_ of Tamarugo stands showed a positive linear relationship with cumulative groundwater depletion.
5. Under water stress, a limitation of the pulvinar movement occurs in Tamarugo trees before they start losing foliage. For this reason, changes in ΔNDVI_mo-mi_ and ΔNDVI_W-S_ are expected to occur before NDVI decreases due to foliage loss, and therefore, ΔNDVI_mo-mi_ and ΔNDVI_W-S_ have potential for early water stress detection.
